# PD-L1 monoclonal antibody-decorated nanoliposomes loaded with Paclitaxel and P-gp transport inhibitor for the synergistic chemotherapy against multidrug resistant gastric cancers

**DOI:** 10.1186/s11671-019-3228-z

**Published:** 2020-03-12

**Authors:** Jinling Yu, Fengli Hu, Qiankun Zhu, Xiaodong Li, Haiyang Ren, Shengjie Fan, Bo Qian, Bo Zhai, Dongdong Yang

**Affiliations:** 1 Department of General Medicine, The Fourth Affiliated Hospital of Harbin Medical University, Harbin, 150001 China; 2 Department of Gastroenterology, The Fourth Affiliated Hospital of Harbin Medical University, Harbin, 150001 China; 3 Department of Surgical Oncology and Hepatobiliary Surgery, The Fourth Affiliated Hospital of Harbin Medical University, Harbin, 150001 China

**Keywords:** Multidrug resistance (MDR), Paclitaxel, Tariquidar, Gastric cancer, Nanoliposomes

## Abstract

Multidrug resistance (MDR) based on ATP-dependent efflux transporters (p-glycoprotein (p-gp)) remains a major obstacle in successful chemotherapy treatment. Herein, we have investigated the potential of PD-L1 mAb-conjugated nanoliposome to serve as a targeted delivery platform for the co-delivery of paclitaxel (PTX) and p-gp specific transport inhibitor (TQD, tariquidar) in drug-resistant gastric cancers. Two drugs, PTX and TQD, were co-loaded in a single vehicle in a precise ratio to enhance the prospect of combination chemotherapeutic effect. Cellular uptake study indicated that PD-PTLP had higher internalization efficiency in PD-L1 receptor overexpressing SGC7901/ADR cells than non-targeted PTLP. Highest synergy was observed at a weight fraction of 1/0.5 (PTX/TQD) and the combination of PTX and TQD resulted in obvious synergistic effect compared to that of individual drugs alone. Our in vitro results showed that TQD was effective in reversing the multidrug resistance in SGC7901/ADR cells. The IC50 value of PD-PTLP was 0.76 μg/ml compared to 6.58 μg/ml and 7.64 μg/ml for PTX and TQD, respectively. PD-TPLP triggered significantly higher levels of reactive oxygen species (ROS) and cell apoptosis compared to that of free PTX or TQD. Furthermore, the in vivo antitumor study showed that the combination chemotherapy of PD-PTLP displayed a significant inhibition of tumor burden of drug-resistant xenograft tumors with significantly higher terminal deoxynucleotidyl transferase dUTP nick end labeling (TUNEL)-positive cells. Furthermore, free PTX resulted in significant increase in the levels of AST and ALT while PD-PTLP insignificantly different compared to that of control indicating the safety index. Overall, we believe that combination of anticancer drug with a p-gp inhibitor could provide a potential direction toward the treatment of drug-resistant gastric tumors.

## Introduction

Present chemotherapeutic treatments based on single drug regimen is far from being perfect and suffers from severe adverse effects at higher doses and simultaneously lead to the development of drug resistance [1]. Past decade has witnessed the high therapeutic efficacy of combinational drug regimen in cancer treatment [2]. Combination of two or more drug has been demonstrated to yield synergistic anticancer efficacy owing to different pharmacological action of combo drugs [3, 4]. However, choosing a right combination of drugs depends on multiple factors including cancer cell type, hydrophilic/hydrophobic drugs, biochemical activities, and pharmacokinetic patterns of the drugs. Among all, combination of drugs is selective to specific types of cancers [5].

Gastric cancer is a global health burden and the second most common cause of cancer-related death worldwide. The prevalence of gastric cancer is high in East Asia such as Japan, Korea, and China with latter reports the highest death rate in the world [6]. On average, 400,000 new cases are registered every year in China and most cases are diagnosed at advanced/later stages [7, 8]. Tremendous progress has been made in the treatment strategies; however, it did not improve the survival rate and resulted in failure in therapy. The treatment failure mainly attributed to the development of chemoresistance and severe toxicity of chemotherapy dose and reoccurrence of gastric cancer episode [9]. Therefore, there is an urgent task to improve the therapeutic efficacy and to overcome the metastasis and reoccurrence of gastric cancer.

Paclitaxel (PTX) is one of the important drugs indicated in gastric cancers treatment [10]. PTX inhibits the cell replication by interfering with the degradation of microtubules thereby led to cell cycle arrest. However, acquisition of multidrug resistance (MDR) is a major hassle between the success of chemotherapy [11, 12]. The ATP-dependent efflux mediated by transmembrane transporters of ATP-binding cassette (ABC) family in which p-glycoprotein (p-gp) is considered the affluent factor is overexpressed in gastric caners [13]. In other way around, PTX serve as a substrate for p-gp receptors wherein drug efflux will reduce the intracellular drug concentration leading to low efficacy and high resistance [14]. In this regard, Tariquidar (TQD) is a potent third-generation p-gp inhibitor and has been reported to reverse the overexpression of p-gp receptors in several cancer cells [15, 16]. However, reports suggested that TQD administration has to be terminated early as it impedes the p-gp function of normal physiological system. P-gp expression is required to maintain the blood-brain barrier (BBB) and removes toxins form the normal tissues [17]. Since p-gp is present in the normal tissues and acts as a barrier against cellular toxins, non-specific inhibition of PTX or TQD could potentially interfere with the normal physiological functions and lead to adverse toxicity. Besides, PTX and TQD are highly lipophilic drugs with limited solubility in aqueous solutions and systemic blood, necessitating the need of a stable drug delivery system which is targeted to the gastric cancers in the body [18].

The drug delivery system (DDS) significantly increased the concentration of encapsulated drugs in the cancer tissues and offered a sustained release of drug for prolonged period of time [19]. In this regard, liposome is one of the widely used drug carriers to improve the therapeutic efficacy in cancers. Liposomes gained increasing importance owing to its biocompatibility, structural surface modifications, hydrophilic/lipophilic drug loading, and high drug loading capacity [20]. The drugs could be stably incorporated in the lipid bilayer of liposome and easily endowed long circulation ability (PEGylation) by enhanced permeation and retention (EPR) effect [21]. Recently, liposome has been reported the ability to retain the multiple drug ratios after intravenous administration [22]. This idea was demonstrated in 2017 after the approval from FDA, Vyxeos® (liposomal formulation) containing ratios of cytarabine:daunorubicin in the treatment of leukemia [23]. Compared with non-targeted formulations, ligand-targeted formulations were very attractive and prospective. In this regard, PD-1 is a cell surface receptor known for the downregulation of immune system and suppresses T cell inflammatory activity. The PD-L1 expression has been reported in 50% of gastric cancer patients making PD-L1 as a targeting receptor for nanoparticle internalization [24, 25]. The PD-L1 monoclonal antibody (mAb) could specifically bind to the extracellular domain of PD-L1 protein and could be an excellent therapeutic strategy to improve the anticancer efficacy [26].

The main aim of present study was to improve the delivery of combinational drugs, PTX and TQD to enhance the anticancer efficacy against gastric cancers. For this purpose, PTX and TQD-loaded PD-L1/nanoliposome was formulated and evaluated for anticancer efficacy at in vitro and in vivo conditions. The in vivo efficacy was evaluated in gastric cancer cell-based xenograft model and immunohistochemistry (IHC) was performed.

## Conclusion

In conclusion, our work realized combination therapy against MDR tumor by programmed delivery strategy. We have demonstrated the potential of combination of PTX and p-gp inhibitor (TQD) in inhibiting the tumor burden of multidrug resistant SGC7901/ADR xenograft tumors. The co-delivery of PTX and TQD in a multifunctional nanocarrier allowed the ratiometric control of co-loaded anticancer drugs, inhibited the p-gp efflux pump, and exhibited the synergistic anticancer efficacy. We believe that combination of anticancer drug with a p-gp inhibitor could provide a potential direction toward the treatment of drug-resistant tumors.

## Materials and methods

### Formulation of PD-L1 mAb-conjugated PTX/TQD-loaded nanoliposomes

The egg phosphatidylcholine (EPC), 1,2-dioleoyl-sn-glycero-3-phosphoethanolamine (DOPE), 1,2-distearoyl-sn-glycero-3-phosphoethanolamine-N-[methoxy(polyethylene glycol)-2000] (DSPE-PEG), and DSPE-PEG2000-maleimide (DSPE-PEG2000-Mal) mixed in a chloroform solution at a molar ratio of 3/2/0.5/0.25 along with PTX and TQD and then organic solvent was evaporated using an rotary evaporator followed by freeze drying for 3 h. The lipid film was hydrated with a 250 mM ammonium sulphate solution maintained at pH 7.0. The large multilamellar liposomes were sonicated for 30 min in a bath-type sonicator (Branson ultrasonicbath, USA) maintained at 65 °C. The liposomes were dialyzed against large volume of distilled water for strictly 1 h to exchange the free drug and initial components. The liposomes were re-dispersed in a phosphate-buffered saline (PBS, pH 7.4). The PD-L1 mAb was conjugated to the liposome by mixing in 8:1 (liposome:mAb) ratio and incubated at 4 °C for 4 h. The PD-L1 mAb will be conjugated to the DSPE-PEG2000-Mal by interacting between sulfhydryl residues on the antibodies to the C-terminal maleimide groups of liposomes. The PD-L1-conjugated liposomes were centrifuged at 10,000 rpm for 10 min and supernatant was removed and re-dispersed in a PBS buffer and stored in 4 °C until further analysis. The amount of PTX and TQD encapsulated in the liposome was evaluated by HPLC method. Agilent Technologies HPLC system model 1260 Infinity equipped with an Agilent Technologies auto sampler and G1315D diode array detector was used in the study. The mobile phase consists of mixture of acetonitrile/water (70:30 v/v) maintained at a flow rate of 1 ml/min. A C18 column (5 μm, 150 × 60 mm, ODS-3) was used to elute the samples and detected at 227 nm. Prior to , PTX/TQD-loaded liposome was dissolved in a acetonitrile and vortexed for 15 min, filtered through 0.45 μm filter, and 10 μl of aliquot was injected in the HPLC column.

### Size distribution and particle morphology analysis

The size distribution and zeta potential of drug-loaded formulations were determined by Malvern zetasizer (UK) at 25 °C. Before the actual experiment, nanoparticle dispersions were diluted 10× with the distilled water and the experiment was performed at a detection angle of 90° in triplicate manner. The morphology of nanoparticles was evaluated by transmission electron microscope (TEM). The nanoparticle dispersions were diluted 10× with the distilled water and placed in a carbon-coated copper grid and dried using IR lamp and then the sample was evaluated by TEM (CM 30, Philips (Eindhoven, The Netherlands)) after staining with uranyl acetate (1% w/v).

### Release profile analysis

The release profile of PTX and TQD from nanoliposome was evaluated by dialysis method. For this purpose, 15 mg of lyophilized powder of PD-L1 mAb-conjugated PTX and TQD-loaded nanoliposomes (PD-PTLP) was dissolved in 1 ml of distilled water and sealed in a dialysis membrane (MWCO 3.5 kDa) and immersed in a 30 ml of respective release buffer maintained at 37 °C. At predetermined time, aliquots of samples were withdrawn and replaced with equal amount of release buffer. The study was continued for 72 h. The samples were filtered through 0.22 μm syringe filter and injected into the HPLC column and evaluated by the method mentioned above.

### Cellular uptake studies

SGC7901/ADR cells were cultured in Dulbecco’s modified Eagle’s medium (DMEM) supplemented with 10% FBS and 100 IU/ml penicillin and 100 μg/ml streptomycin under the condition of 5% CO_2_ atmosphere at 37 °C. A confocal laser scanning microscope (BX61WI; Olympus, Tokyo, Japan) was used to evaluated the cellular distribution and cellular uptake of PTLP and PD-PTLP in SGC7901/ADR cells. To attain this purpose, 1 × 10^5^ cells were seeded in each well of 12-well plate and incubated for 18 h. The cells were then exposed with PTLP and PD-PTLP and incubated for 3 h. The cells were washed three times with cold PBS and fixed with 4% paraformaldehyde (PFA) for 10 min. The cells were again washed with PBS and stained with 4,6-diamidino-2-phenylindole (DAPI) for 10 min. Finally, cells were carefully washed and observed under CLSM. The competitive experiment on the uptake of PD-PTLP was performed by pretreating the cells were free PD-L1 mAb for 30 min and washed. The cells were divided into two groups, one treated with free PD-L1 mAb and other non-treated with free PD-L1 mAb. The cells were incubated with PD-PTLP and incubated for 3 h and same method was followed for the evaluation of cellular uptake analysis.

### Protein expression by Western blot assay

The SGC7901/ADR cells were seeded in 6-well plate at a seeding density of 3 × 10^5^ cells/well and incubated for 18 h. The cells were treated with different formulations (PTX, TQD, PTLP, PD-PTLP) and incubated for 24 h. Cells were washed and extracted with stripping buffer and lysed using a standard lysis buffer (Santa Cruz Biotechnology, Santa Cruz, CA, USA). The cells were centrifuged at a speed of 12,000 rpm for 15 min and supernatant was collected and protein quantification was performed using BCA protein assay (Beyotime). Equal amount of proteins was loaded in 8% SDS-PAGE gel and then transferred to a nitrocellulose membrane (EMD Millipore, Billerica, MA, USA). The membrane was blocked with 5% skim milk for 1 h to inhibit the non-specific binding sites. The membrane was incubated with primary antibodies (p-gp and GAPDH, 1:1000, Abcam, MA, USA) at 4 °C overnight. The membrane was washed with TBST and again incubated with secondary antibodies of horseradish peroxidase-labeled goat anti-rabbit or -mouse antibodies (rabbit or mouse, 1:10,000, Abcam, MA, USA) at room temperature. The membranes were washed again with TBST. The blots were visualized under an enhanced chemiluminescence method (EMD Millipore).

### In vitro cytotoxicity analysis

The cytotoxic effect of individual drugs and formulations were evaluated by MTT assay. Briefly, cancer cells were layered at a density of 1 × 10^4^ cells/well in a 96-well plate and incubated for 18 h. The cells were then treated with free PTX, TQD, PTLP, and PD-PTLP, respectively for 24 h. Next, cells were carefully washed and added with 15 μl of 5 mg/ml MTT solution and subjected for 3 h of additional incubation and then 100 μl of DMSO was added to extract the formazan crystal. The resulting absorbance was measured at 570 nm using an automated microplate reader. Cell viability is calculated by OD of test group/OD of control × 100%. The combination index was evaluated by Calcusyn^TM^ software. All the experiments were performed at triplicate.

### In vitro apoptosis and reactive oxygen species analysis

For apoptosis assay, cancer cells were layered at a density of 2 × 10^5^ cells/well in a 12-well plate and incubated for 18 h. The cells were then treated with free PTX, TQD, PTLP, and PD-PTLP, respectively for 24 h. The cells were extracted by stripping and centrifuged and pellets were re-dispersed in 100 μl of binding buffer. The cells were co-stained with a combo of 5 μl of Annexin-V/FITC and 2.5 μl of PI working solution and incubated for 15 min. The stained cells were analyzed by flow cytometer using a BD FACS Calibur (BD Biosciences, CA, USA). The Annexin-V and PI represents the early apoptosis indicator and late apoptosis indicator based on the structural components of live and dead cells, respectively.

The 2,7-dichlorofluorescin diacetate (DCFH-DA) was used as a probe for the reactive oxygen species (ROS) analysis. For quantitative analysis, 1 × 10^4^ cells/well were seeded in a 96-well black-bottomed plate and incubated for 18 h. The cells were then treated with free PTX, TQD, PTLP, and PD-PTLP, respectively for 24 h. The cells were washed with PBS buffer and then incubated with 1 ml of DCFH-DA solution as per the manufacturer’s guideline for 30 min. Followed by cells lysed and centrifuged at 10,000 rpm for 15 min. The resulting supernatant was transferred to a new 96-well plate and the fluorescence was measured at 485 nm using an automated microplate reader. Simultaneously, separate set of cells were processed in the similar manner and images were observed using fluorescence microscope (Nikon A1, Japan).

### Antitumor efficacy of PD-PTLP in xenograft model

The antitumor efficacy study was performed in BALB/c nude mice and was obtained from Laboratory Animal Center, The Fourth Affiliated Hospital of Harbin Medical University, Harbin. All animal experiments were conducted in accordance with the national standards for laboratory animal’s quality. The experiments were strictly performed in accordance with the Regulations for Experimental Animals Committee guidelines of The Fourth Affiliated Hospital of Harbin Medical University, Harbin. The animals were subcutaneously injected with 1 × 10^6^ SGC7901/ADR cells in 150 μl of culture media in the right flank of the mice. The tumors were allowed to grow until 100 mm^3^ before the actual experiment. The mice were equally divided into five groups with eight mice in each group. The individual dose of PTX and TQD were fixed at 5 mg/kg, whereas combination used a total dose of 5 mg/kg. The tail vein injection was every third day, in total, three injections were performed. At predetermined days, tumor volume and body weight were measured. The tumor volume was calculated by measuring the longest diameter and shortest diameter of the tumors using a digital caliper. Tumor volume (*V*) = ½ × length × width (mm)^2^. The mice were sacrificed at the end of the study and the tumor was extracted and weighed. The tumor was subjected to immunohistochemical (IHC) analysis. The tumors were extracted, sliced into a thin piece, and fixed in 10% formalin solution. The tumors were embedded in a paraffin wax and then performed the TUNEL assay as per the manufacturer’s guidelines.

### Serum biochemical analysis

The mice were administered with respective formulations; 24 h later, mice were sacrificed and blood samples were collected from the control as well as test treated animal groups. The serum is separated from the whole blood and stored in − 80 °C until further analysis. Serum biochemical analysis was conducted to assess the performance of liver. Aspartate transaminase (AST) and alanine transaminase (ALT) were measured to evaluate liver function. All measurements were performed according to the procedures of the biochemical kit assay.

## Results and discussion

### Preparation and characterization of PTX/TQD-loaded PD-L1-conjugated nanoliposomes

**Fig. 1:**
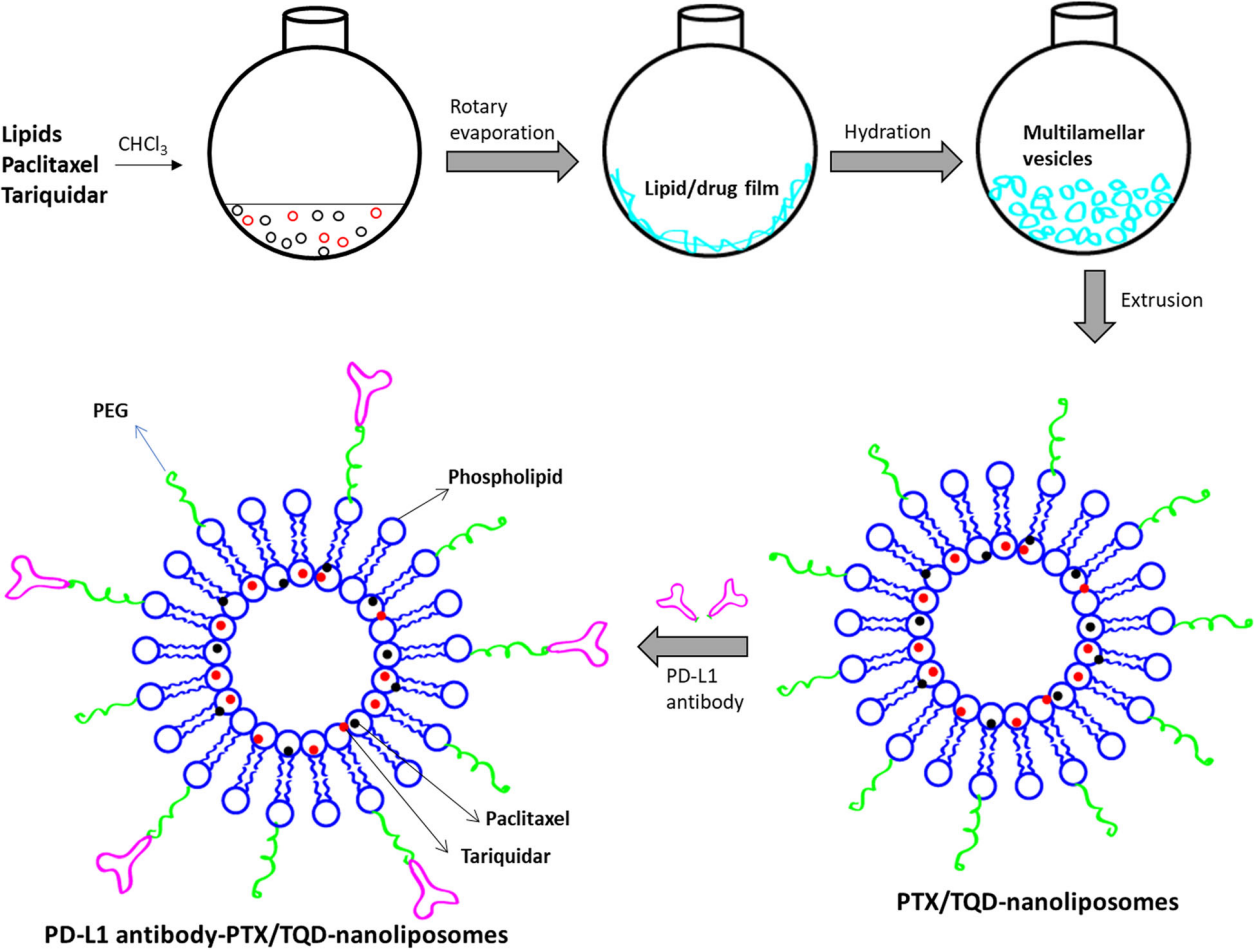
Schematic illustrations of loading of paclitaxel and tariquidar in PD-L1 monoclonal antibody (mAb)-surface conjugated nanoliposomes. The liposome was prepared by hydration of thin-lipid film and sonicated to form the drug-loaded nanoliposomes

The PTX has been widely used in the clinics and primarily indicated in the treatment of gastric cancers. However, majority of patients apparently suffers from poor therapeutic response due to multidrug resistance (MDR) of the gastric cancers. An increase in the dose of PTX resulted in increased systemic toxicity thereby MDR became a major obstacle in the successful cancer therapy treatment. Among plethora of MDR mechanisms, p-gp-mediated drug efflux is considered to be primarily responsible for the drug resistance in cancer cells [27]. Therefore, in this study, we have used a second drug (TQD) as a p-gp inhibitor to overcome the MDR phenomenon in the cancer cells and improve the anticancer efficacy of PTX. We have carefully studied the weight fraction of two drugs in which they exhibit the synergistic effect. In order to maximize the anticancer effect, it is important to co-deliver the multiple drugs in a single nanoparticle system. Delivery of two drugs (PTX and p-gp inhibitor) simultaneously will allow the efficient suppression of drug efflux mechanism and increase the prospect of increased intracellular concentrations in the cancer cells [28]. For this purpose, in this study, we have loaded two drugs in the lipid bilayer of nanoliposomes which are considered extremely stable in the systemic circulations (Fig. 1). To achieve the enhanced tumor-specific targeting, nanoliposomes were surface conjugated with PD-L1 mAb. The maleimide group present in the nanoliposome will bind with the thiol group of PD-L1 mAb and form a stable covalent bond. But a possible limitation of maleimide conjugation is that the reaction is reversible: the product might undergo retro–Michael addition reactions with biological thiols in plasma which may lead to the release of the maleimide. However, we have overcome such reaction by maximum surface conjugation of antibody on the maleimide-liposome. It has been reported that tumor-targeting ligand will specifically delivery the therapeutic load in the tumor tissues and avoid unnecessary side effects in the normal tissues. Earlier, Patel et al. reported that incorporation of p-gp inhibitor with PTX could overcome the MDR in the ovarian cancer cell at in vitro conditions [11]. Similarly, Zou et al. and Zhang et al. reported that the PTX cytotoxicity against SKOV-3TR and A2780-Adr multidrug-resistant cells increased significantly in the presence of Tariquidar. However, in these studies, either physical combination of PTX + TQD was used or non-targeted carrier was developed [29, 30]. Importantly, all these studies have been performed only at in vitro conditions. The present study focused on designing the targeted nanocarrier using a relatively new class of targeting agent, PDL1 antibody. Besides, the present study demonstrated the efficacy of PTX + TQD in xenograft tumor model and also assessed the blood parameters to relate with the systemic toxicity.

**Fig. 2:**
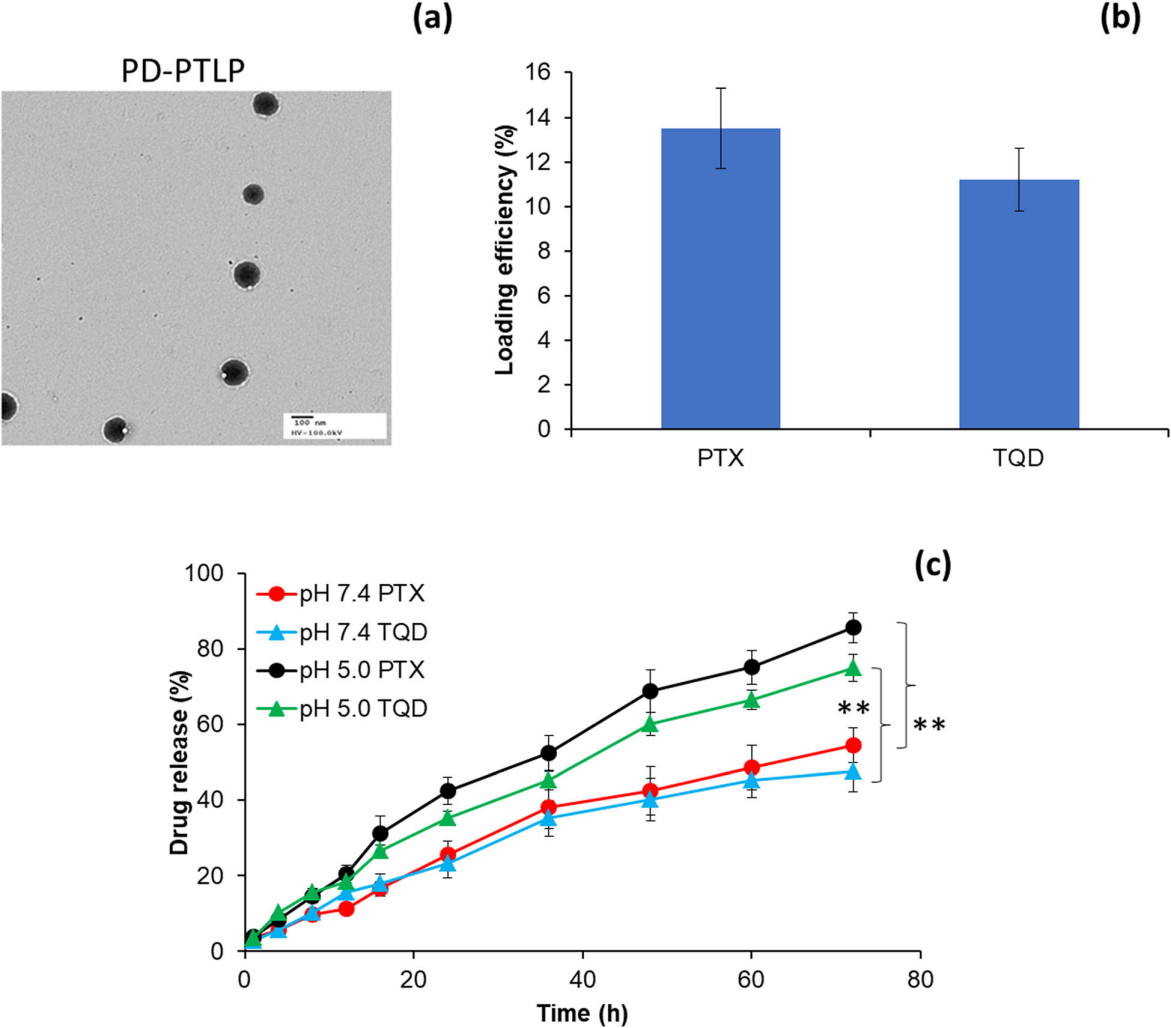
Physicochemical characterization of PTX/TQD-loaded PD-L1-conjugated nanoliposome. **a** Morphology analysis of PD-PTLP using transmission electron microscope (TEM). **b** Drug loading capacity of PD-PTLP. **c** In vitro release of PTX and TQD from PD-PTLP at pH 7.4 buffer and pH 5.0 buffer conditions at 37 °C. **p < 0.01 is the statistical difference in drug release between pH 7.4 and pH 5.0 buffers

The average particle size of PTLP was 135.6 ± 1.26 nm while it increased to 168.59 ± 1.34 nm after conjugation with PD-L1 mAb (PD-PTLP). The particle size increased due to the large molecular weight of PD-L1 mAb; nevertheless, overall size less than 200 nm and spherical shape was the point worth noting (Fig. 2a). Nanoparticles size less than 200 nm will allow the higher accumulation in tumor tissues owing to enhanced permeation and retention (EPR) effect. Besides, the presence of PEG will allow the prolonged blood circulation time in the systemic circulation. The zeta potential of PD-PTLP was 22.1 ± 1.21 mV which will not allow any non-specific binding to the blood components. PD-PTLP exhibited a high entrapment efficiency of ~ 95% for both the drugs (PTX and TQD). PD-PTLP also exhibited a high drug loading of 12–14% w/w for PTX and TQD, respectively (Fig. 2b).

### In vitro drug release

The release behavior of PTX and TQD from PD-PTLP was studied in pH 7.4 and pH 5.0 conditions at 37 °C. As shown in Fig. 2c, a controlled release of drugs (PTX and TQD) was observed from the PD-PTLP throughout the study period (72 h). The release of drug from the thick bilayer of nanoliposome could be responsible for the slow and sustained release of two drugs from the PD-PTLP. It must be noted that no significant difference was observed in the release pattern of PTX and TQD in pH 7.4 and pH 5.0. At longer time point, significant difference is release was observed in different pH conditions. It is worth noting that no pH-responsive elements were added in the nanoliposome and higher drug release in acidic conditions might be attributed to the higher diffusion at lower pH. For example, ~ 85% of PTX released in pH 5.0 conditions compared to ~ 55% of drug release at physiological pH environment. A similar pattern of rapid release of small molecules at acidic conditions and slower release in basic pH conditions have been demonstrated by other researchers. Nevertheless, relatively low drug release in pH 7.4 conditions might reduce the unnecessary systemic side effects and prolong the systemic circulation while higher drug release in pH 5.0 might benefit the higher therapeutic efficacy in the tumor tissues.

### In vitro cellular uptake analysis

**Fig. 3:**
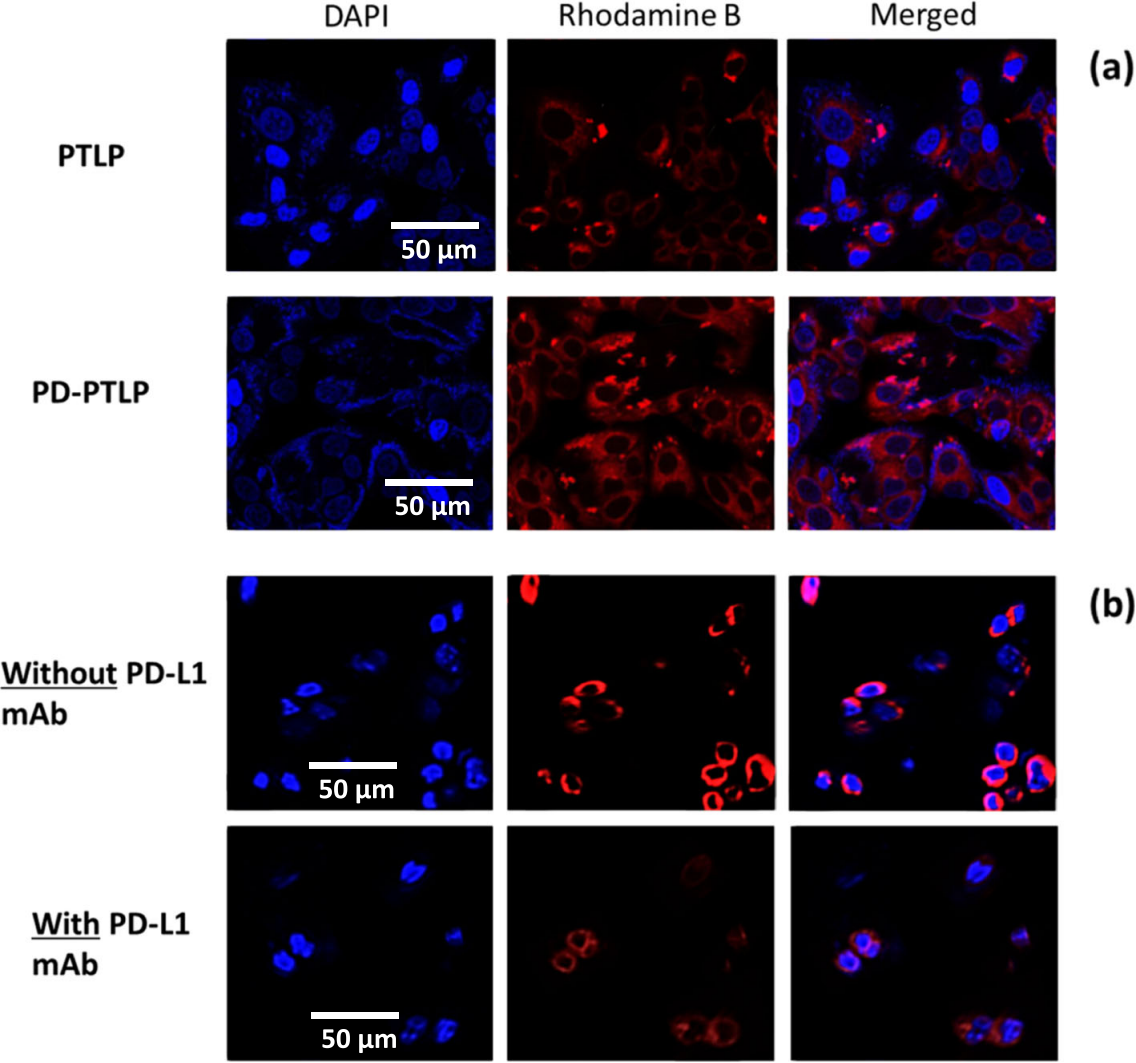
**a** Confocal laser scanning microscopy (CLSM) images of SGC7901/ADR cells after incubation with PTLP and PD-PTLP for 3 h; CLSM images of SGC7901/ADR cells pretreated with/without PD-L1 mAb after incubation with PD-PTLP for 3 h. Rhodamine B was used as a fluorescent tracker and DAPI was used to stain the nucleus of the cancer cells

The delivery efficiency of targeted (PD-PTLP) and non-targeted (PTLP) nanoliposomes were tested in SGC7901/ADR. The cellular uptake was evaluated using rhodamine-B as a fluorescence tracker. Rhodamine-B is the commonly used fluorophore with no cell biological interactions. Nucleus was stained with blue-colored DAPI and red color originated from the nanoparticles. The CLSM data clearly reveal that PD-PTLP exhibited a strong red fluorescence in the cancer cells compared to that of non-targeted PTLP. A higher red fluorescence in PD-PTLP-treated cancer cells attributed to the higher nanoparticle internalization (Fig. 3a). The outcome of the CLSM data indicates that the PD-L1 receptor expressed on the cell membrane was recognized by PD-L1 mAb conjugated on the nanoliposome surface. A non-specific or passive uptake mechanism was evident in the PTLP-treated cancer cells. The PD-L1 target specificity was further confirmed by PD-L1 mAb pretreatment experiment. The SGC7901/ADR cells were pretreated with PD-L1 mAb and incubated for 30 min. The cells were then exposed with PD-PTLP and PTLP and incubated for 3 h. As shown (Fig. 3b), cells pretreated with PD-L1 mAb showed significantly less red fluorescence compared to that of non-treated cells indicating that PD-L1 mAb was consumed by the surface-expressed receptors and no additional receptors were available for binding and internalization resulting in less uptake of nanoparticles and less internalization. These CLSM clearly revealed the targeting specificity of PD-PTLP in SGC7901/ADR cancer cells.

### Dual drug-loaded nanoliposomes enhances antiproliferative effect

**Fig. 4:**
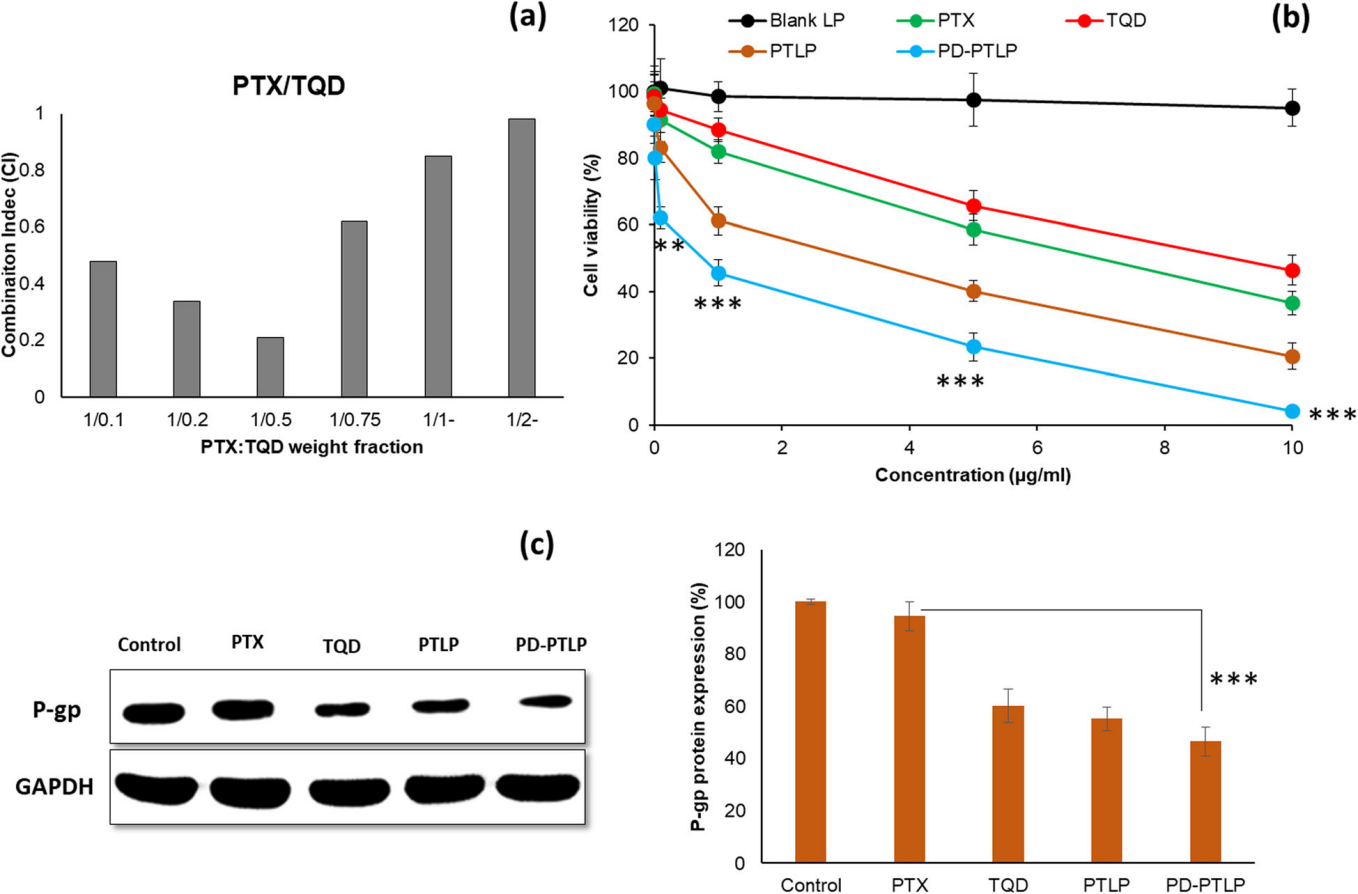
**a** Combination index (CI) of different weight fractions of PTX and TQD in SGC7901/ADR cells. The combination index was evaluated by Calcusyn^TM^ software. **b** In vitro cell viabilities of SGC7901/ADR cells after treatment with various concentrations of free PTX, TQD, PTLP, and PD-PTLP for 24-h incubation period. **c** Western blot analysis of p-gp expression in SGC7901/ADR cells after treatment with respective formulations. **p < 0.01 and ***p < 0.001 is the statistical difference between free PTX and PD-PTLP-treated group

For combination therapy, different ratios of two drugs (PTX and TQD) were used to establish the extent of synergistic or additive effect on the resistant gastric cancer cells. To calculate the isobologram and combination index (CI), CalcuSyn software (Biosoft, Version 2.1) was used. The isobologram plot could be explained based on the Chou-Talalay equation. The CI values are characterized by synergistic (CI < 0.9), additive (CI = 1), and antagonistic (CI > 1). As shown, all combination ratio of PTX and TQD revealed a CI value of < 1 signifying a synergistic mechanism of action (Fig. 4a). To be specific, highest synergy was observed at a weight fraction of 1/0.5 (P/T) while the level of synergy decreased with the increase in the TQD weight fraction indicating the importance of presence of two drugs in specific weight fraction. Too low and too high concentration of TQD in a combination regimen did not yield the best synergistic effect. For all the in vitro experiments, we have used P/T = 1/0.5 ratio in the study.

The in vitro cytotoxic effect of individual as well as combinational drugs was determined by MTT protocol after 24-h incubation. As shown in Fig. 4b, blank NP did not have any effect on the cell viability ruling out the possibility of any interference in the final outcomes. The monotherapy with PTX and TQD though showed a concentration-dependent cytotoxic effect in the resistant gastric cancer cells; it fell short of appreciable effectiveness in treatment. The anti-proliferative effect of single agent was remarkably improved when combined with the second drug encapsulated in a nanoliposome. The combination of PTX and TQD resulted in obvious synergistic effect compared to that of individual drugs alone. Moreover, PD-L1 mAb-conjugated nanoliposome (PD-PTLP) exhibited the strongest anti-proliferative effect indicating the influence of the targeting ligand on the nanoparticle surface. This enhanced cell killing in the PD-L1-targeted treatment group might be attributed to the high cellular internalization of PD-L1-targeted nanoliposomes by SGC7901/ADR cells consistent with the cellular uptake analysis. The IC50 value of PD-PTLP was 0.76 μg/ml compared to 6.58 μg/ml and 7.64 μg/ml for PTX and TQD, respectively. A ten-fold decrease in IC50 value of PD-PTLP clearly indicates that resistance to PTX in p-gp overexpressing SGC7901/ADR was reversed by TQD. Our in vitro results showed that TQD was effective in reversing the multidrug resistance in SGC7901/ADR cells. Results also showed that nanoliposomes retained the pharmacological actions of encapsulated drugs and released the drug in a controlled manner in the cancer cells. The combination therapy with PTX and TQD enhanced the anticancer efficacy with increased synergistic activity, outperforming the minimal advantages of monotherapy and possible associated side effects. Overall, combination treatment of PTX with an effective p-gp inhibitor in nanoliposome could be a promising strategy to overcome MDR and treat gastric cancers.

In order to evaluate the molecular mechanism, Western blot analysis was performed on SGC7901/ADR. As shown (Fig. 4c), PTX did not have any effect on the p-gp protein expression while on the contrary, TQD significantly downregulated the p-gp expression confirming its pharmacological role as a p-gp inhibitor. Interestingly, combination drug-based PTLP and PD-PTLP showed insignificant difference in protein expression compared to that of TQD-treated cancer cells. The result demonstrated the advantage of loading PTX and TQD (P-gp inhibitor) together in a single nanocarrier system. The Western blot result could be corroborated with the cell viability results where combination of PTX + TQD reversed the MDR and exhibited higher anticancer efficacy in gastric cancer cells.

### Apoptosis analysis by flow cytometer

**Fig. 5:**
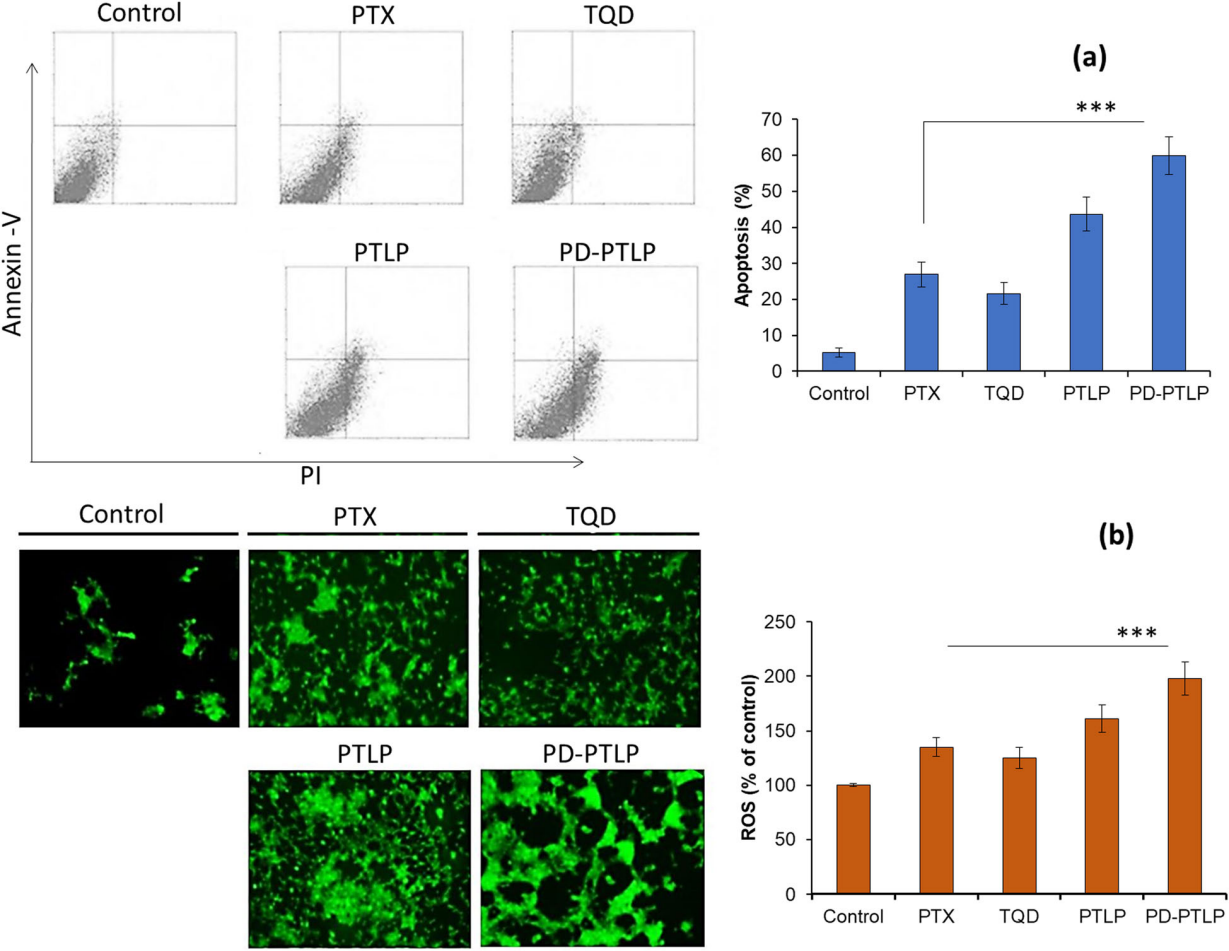
**a** Apoptosis assay of SGC7901/ADR cells after staining with Annexin V/PI combo using flow cytometer. The cells were treated with a fixed concentration of 2 μg/ml. **b** Reactive oxygen species (ROS) analysis of SGC7901/ADR cells using 2,7-dichlorofluorescin diacetate (DCFH-DA) as a probe. ***p < 0.001 is the statistical difference between free PTX and PD-PTLP-treated group

Apoptosis analysis of individual formulation was evaluated by Annexin V-FITC/PI staining method using flow cytometer. Results of apoptosis are presented in Fig. 5a. A shown, control cells did not show any sign of apoptosis, whereas free PTX and TQD exhibited obvious increase in the apoptosis cells. Combination drug-based PTLP showed two-fold higher apoptosis compared to that of individual drugs indicating the synergistic anticancer effect of the formulations. More importantly, PD-PTLP showed the highest apoptosis of cancer cells with around 60% under apoptosis region. Enhanced apoptosis effect of PD-PTLP was attributed to higher internalization of dual-drug-loaded nanocarriers and synergistic potential of PTX and TQD in a ratiometric manner. The p-gp silencing effect of TQD in combinational regimen enhanced the anticancer effect of PTX in the cancer cells. The apoptosis rate of individual drug was in the range between 20 and 25% while around 60% of apoptosis cells were observed for PD-PTLP-treated cells. The PD-PTLP induced more apoptosis than free drugs and non-targeted liposomes, suggesting that the PD-L1 could deliver PTX/TQD more efficiently to induce apoptosis of the SGC7901/ADR cells.

### Intracellular ROS level determination

We have explored the ability of individual drug and dual drug to affect the redox state of the cancer cells cell by evaluating the level of reactive oxygen species (ROS) in gastric cancer cells. ROS levels in cancer cell were evaluated by DCFH-DA (green fluorescence). Quantitative ROS data are presented in Fig. 5b. As shown, non-treated cells did not have any sign of ROS; however, PTX or TQD did induce appreciable levels of ROS generation. Importantly, TPLP and PD-TPLP triggered a significantly higher levels of ROS compared to that of free PTX or TQD or non-treated control cells. A remarkably higher ROS indicates the potential of PD-TPLP to promote higher apoptosis. Microscopic images corroborate with the quantitative results with brightest and higher intensity green fluorescence compared to untreated or free PTX or TQD treated cancer cells. The higher intensity of green fluorescence is an indication of higher ROS production. Oxidative stress such as ROS is considered to be an important indicator of cellular cytotoxicity. Studies have shown that induction of ROS induce a scores of physiological events including DNA damage, inflammation, and cell apoptosis.

### Combination of PTX and TQD inhibited growth in drug-resistant tumors

**Fig. 6:**
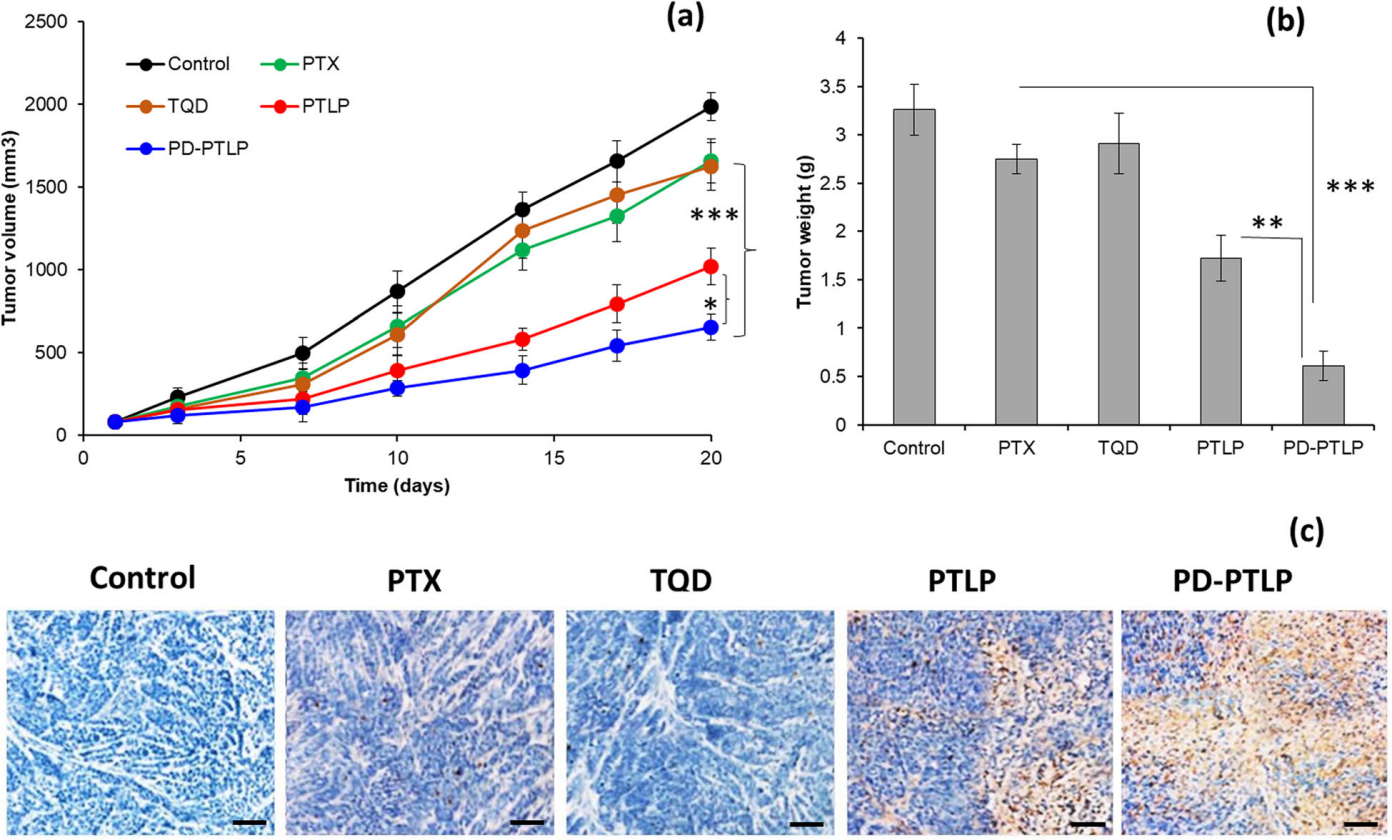
In vivo antitumor efficacy of different formulations against multidrug resistant (MDR) SGC7901/ADR tumors; **a** tumor volume, **b** tumor weight analysis, and **c** TUNEL assay of tumor tissues. The mice were administered with a fixed dose of 5 mg/kg with duration of three times for three administrations. Apoptotic cells wells were evaluated by TUNEL assay. *p < 0.05 and **p < 0.05 (PTLP vs. PD-PTLP), ***p < 0.001 (PTX vs. PD-PTLP)-treated group.

Finally, therapeutic efficacy and toxicity parameters of formulations were investigated on drug-resistant SGC7901/ADR xenograft tumor model (Fig. 6a). The drugs were intravenously administered at a fixed dose of 5 mg/kg for every 3 days with a total of three injections. The PTX/TQD was administered at a fixed weight fraction of 1/0.5. On the expected line, free PTX and free TQD did not show any inhibitory effect on the growth of MDR tumors, suggesting the fact that the SGC7901/ADR cells manifest drug tolerance on the proliferation of MDR tumors. P-gp inhibitor (TQD) though efficient in inhibiting the drug efflux pumps however does not convert into improved therapeutic outcome. In comparison, combination of PTX + TQD (PTLP) displayed a significant inhibition of growth of drug-resistant tumors. The best antitumor efficacy was observed with PD-PTLP which was three-fold effective compared to control, 2.5 compared to free drugs, and approximately two-fold effective in reducing the tumor burden compared to non-targeted formulations (*p* < 0.05; *p* < 0.001). The final tumor volume of control, free PTX, TQD, PTLP, and PD-PTLP was ~ 2000 mm^3^, ~ 1650 mm^3^, 1625 mm^3^, ~ 1000 mm^3^, and ~ 650 mm^3^, respectively. Free drugs were slightly effective during the initial time point; however, they grew the same as that of non-treated control group. Results clearly reveal the potential of combination of PTX + p-gp inhibitor as a unique strategy to effectively control the tumor burden. The extensive tumor suppression in PD-PTLP clearly suggests the greater antitumor efficacy of the targeted formulations group. The tumors were extracted and weighed; tumor weights were consistent with the tumor volume data (Fig. 6b). PD-PTLP-treated mice group showed the smallest tumor compared to any other formulation-treated group (*p* < 0.001). To further verify the inhibitor effect of individual tumors, tumors were subjected to TUNEL assay (Fig. 6c). As shown, PD-PTLP showed the large swaths of apoptosis staining compared to non-treated control or free drugs. PD-PTLP showed apparent apoptosis traits with disorganized cell arrangements. The prominent tumor killing effect of PD-PTLP displays the greater cancer cell inhibition in drug-resistant tumor cells. The excellent efficacy of PD-PTLP was mainly attributed to the presence of targeted ligand (PD-L1 mAb) which binds with the respective receptors and increases the intracellular concentrations. The presence of combination regimen and release in a controlled manner for prolonged time also contributed for its enhanced efficacy. In addition, dense hydrophilic PEG surface corona might offer excellent physical stability to the particles and could potentially avoid the unnecessary protein absorption and avoid rapid clearance. The long circulation and nano-scaled size in turn benefit the higher accumulation of particles in the tumor tissues [31–33].

### Systemic toxicity analysis

**Fig. 7:**
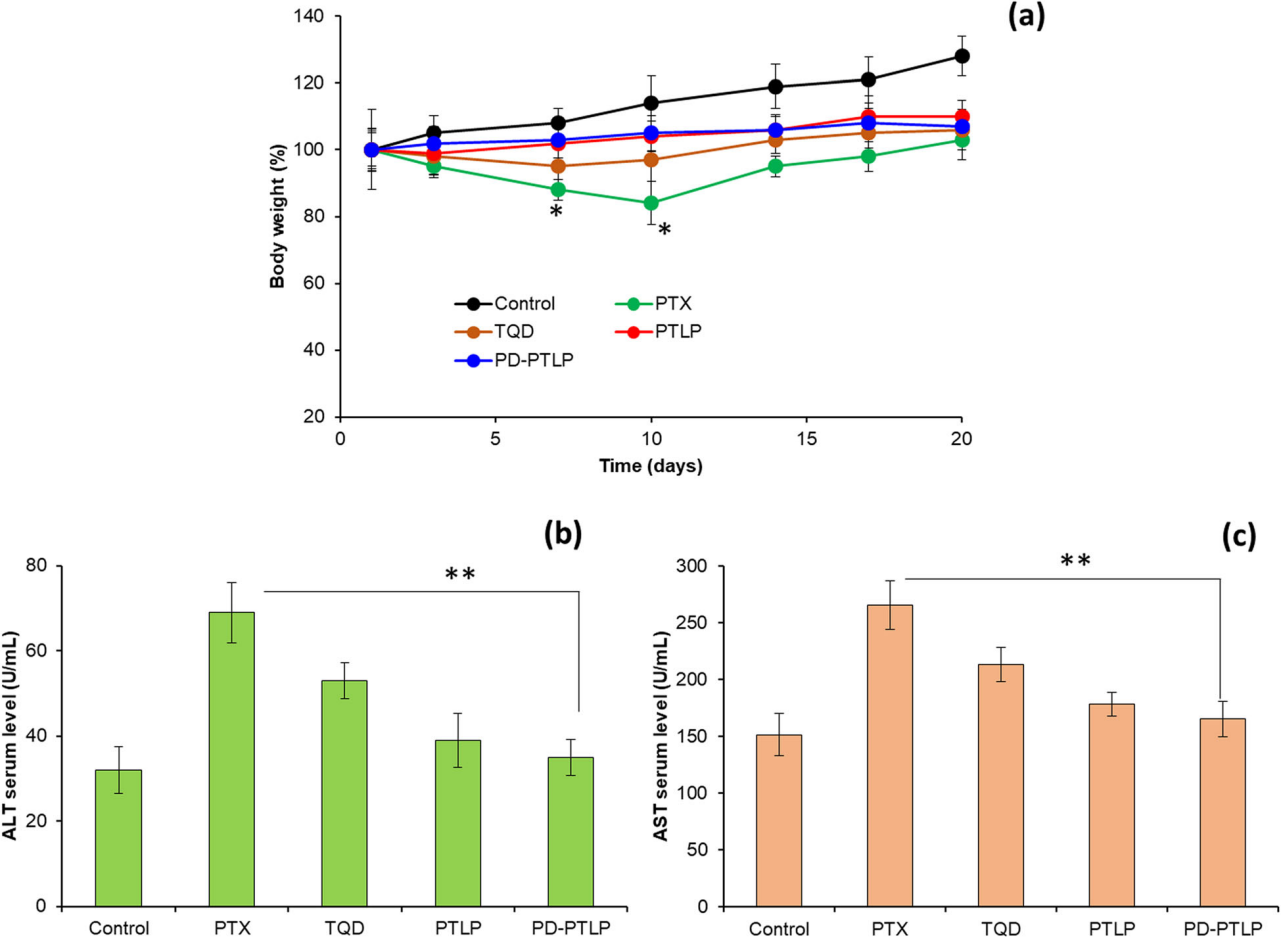
Systemic toxicity analysis of individual formulations in SGC7901/ADR tumors; **a** mice body weight analysis; **b**, **c** blood biochemical evaluation of serum levels of AST and ALT as a systemic toxicity parameters. *p < 0.05 and **p < 0.01 is the statistical difference between free PTX and PD-PTLP treated group

The change in body weight is a good indicator of systemic toxicity. As shown (Fig. 7a), mice treated with free PTX shed ~ 20% of body weight on day 10 which gradually decreased to regain the original body weight toward the end of the study. Loss of more than 5% of body weight is considered to cause severe internal toxicity and 20% of body weight loss is considered a significant adverse effect of free drugs [31]. On the contrary, PTLP or PD-PTLP did not cause any such loss of body and the mice remain healthy throughout the study period indicating the safety index of the nanoliposomes. Delivery system with no systemic toxicity and enhanced antitumor efficacy is considered to be highly effective in tumor treatment. The safety of nanoparticles was further studied by measuring plasma levels of enzymes. Plasma levels of aminotransferases (AST and ALT) are measured following the 24-h administration of respective formulations (Fig. 7b, c). As shown, free PTX resulted in significant increase (*p* < 0.01) in the levels of AST and ALT while PD-PTLP and PTLP were insignificantly different compared to that of non-treated control. AST is released in serum upon organ damage such as heart, kidney, or liver while ALT is specifically released in case of liver injury [34]. The levels of AST and ALT serves as a specific indicator of organ damage and in this regard PD-PTLP showed to be a safe carrier.

## Data Availability

All data generated or analyzed during this study are included in this published article and its supplementary information files.

## Abbreviations

EPR: Enhanced permeation and retention effect
PD-PTLP: PD-L1 mAb-conjugated PTX and TQD-loaded nanoliposomes
P-gp: P-glycoprotein
PTLP: PTX and TQD-loaded nanoliposomes
PTX: Paclitaxel
TQD: Tariquidar
